# Prevalence of dysmenorrhea and its effect on quality of life among a group of female university students

**DOI:** 10.3109/03009730903457218

**Published:** 2010-04-07

**Authors:** Alaettin Unsal, Unal Ayranci, Mustafa Tozun, Gul Arslan, Elif Calik

**Affiliations:** ^1^Eskisehir Osmangazi University, Public Health Department, Meselik-EskisehirTurkey; ^2^Osmangazi University, Medico-Social Center, Meselik-EskisehirTurkey; ^3^Ministry of Health, Community Health Center, Yenikent-EskisehirTurkey; ^4^Eskisehir Osmangazi University, Eskisehir Vocational College of Health Services, Meselik-EskisehirTurkey; ^5^Dumlupinar University, College of Nursing, KutahyaTurkey

**Keywords:** Dysmenorrhea, female students, quality of life, SF-36, VAS

## Abstract

The objective was to evaluate the prevalence of dysmenorrhea and determine its effect on health-related quality of life (HRQoL) among a group of female university students. This cross-sectional study was conducted between 15 March and 15 April 2009 at Dumlupinar University, Kutahya, Health High School, Western Turkey. The study group included 623 female students. The severity of dysmenorrhea was determined with a 10-point visual analog scale. The Short Form-36 (SF-36) form was used to determinate HRQoL. Chi-square test, Student's *t* test, and logistic regression and variance analyses (ANOVA) were used for statistical analyses. The average age of the study group was 20.8 ± 1.8 years (range 17–30). Prevalence of dysmenorrhea was found to be 72.7% and was significantly higher in coffee consumers, females with menstrual bleeding duration ≥7 days, and those who had a positive family history of dysmenorrhea when compared to the others (*P* < 0.05, for each one). By multivariate analysis, coffee consumption (OR 2.084), menstrual bleeding duration ≥7 days (OR 1.590), and positive family history of dysmenorrhea (OR 3.043) were important risk factors for dysmenorrhea. Except for social functioning, role–emotional, and mental health domains, the SF-36 points received from the other domains were higher in females with dysmenorrhea (for each one *P* < 0.05). With the exception of the scores received from physical functioning and role–emotional domains, the scores received from the other domains of the SF-36 scale showed a decrease with increasing severity of dysmenorrhea (*P* < 0.05, for each one). Dysmenorrhea is a common health problem, having negative effects on the HRQoL among university female students.

## Introduction

Dysmenorrhea may be categorized into two distinct types: primary and secondary. Primary dysmenorrhea is defined as painful menses in women with normal pelvic anatomy, usually beginning during adolescence (1). Secondary dysmenorrhea is menstrual pain associated with underlying pathology, and its onset may be years after menarche. It can be caused by any of a dozen or so disorders such as endometriosis, pelvic inflammatory disease, intra-uterine devices, irregular cycles or infertility problems, ovarian cysts, adenomyosis, uterine myomas or polyps, intra-uterine adhesions, or cervical stenosis.

Dysmenorrhea is characterized by crampy pelvic pain beginning shortly before or at the onset of menses and lasting 1–3 days (2). Some 2–4 days before menstruation begins, prostaglandins proceed into the uterine muscle where they build up quickly at menstrual onset and act as smooth muscle contractors that aid in the expulsion of the endometrium (3).

There is a wide variation in the estimate of dysmenorrhea from studies around the world reporting a range between 28% and 71.7% (4,5). In similar studies from Turkey, the prevalence of dysmenorrhea has been reported to be between 58.2% and 89.5% (6,7).

Studies on the prevalence of menstrual pain have shown that many factors are related to this disorder. These factors include a younger age, low body mass index (BMI), smoking, early menarche, prolonged or aberrant menstrual flow, perimenstrual somatic complaints, pelvic infections, previous sterilization, somatization, psychological disturbance, genetic influence, and a history of sexual assault influencing the prevalence and severity of dysmenorrhea (8,9).

Emotional and behavioral problems may exacerbate menstrual cycle problems and dysmenorrhea. For example, depression and/or anxiety symptoms are reported to have an impact on menstrual cycle function and dysmenorrhea. Due to the negative effects of dysmenorrhea on an individual's psychological status, health-related quality of life (HRQoL) may be disrupted among adolescent women (10). Furthermore, dysmenorrhea is a common cause of sickness absenteeism from both classes and work by the female student community (11).

The aim of this study was to evaluate the prevalence of dysmenorrhea and its effect on HRQoL among female students of a university situated in western Turkey.

## Methods

### Setting

Kutahya is a semi-rural province situated in the western part of Turkey, with a population of 565,884. The socio-economic level of the city is average compared with other cities of the country. There are significant disparities in the socio-economic characteristics of the quarters of the city. The city includes one university and has a cosmopolitan structure in terms of student population. The university where the study was conducted, Dumlupinar University, has 19 faculties and colleges with about 30,000 students.

### Sampling

This cross-sectional study was conducted between 15 March 2009 and 15 April 2009 in the Health College giving education at the Dumlupinar University, a public university located in the west of Turkey. The number of the students studying in that college was 946, most of them (*n* = 664, 70.2%) girls. The study was performed on a total of 623 students who agreed to participate in the research. Of the 664 students, 41 were excluded from the survey due to: unwillingness to participate in the research (*n* = 9), and not being at classes at the time of the study (*n* = 32). The remaining 93.8% (623/664) women constituted the study group.

### Procedures

All 623 students surveyed at school completed the questionnaires and inventories during a class period. After distributing the questionnaires to students at the school, they were informed of how the questionnaires were to be filled in and then were requested to make a choice applicable to themselves. The students completed questionnaires and inventories in the presence of a member of the research team. The data collected was self-reported by the students. All subjects (*n* = 623) were told that participation in the investigation was strictly voluntary, and that the data collected would not be used for anything except for this research study, and they were given the questionnaire and inventory to complete. The duration for completing the questionnaire and inventory was between 35 and 40 minutes per subject. The principal investigators met daily with the data collectors to ensure the quality of data collected.

### Development of the questionnaires

The questionnaire, prepared with reference to previous studies in the literature (7,12,13), included three parts. In the first part of the questionnaire, students were asked to state their socio-demographic and medical characteristics, and dysmenorrheal status and habits. The second part of the questionnaires included visual analogue scale (VAS) questions and multidimensional scoring system (MSS) (Table I) to assess the severity of dysmenorrhea (14–16), and the last part included the questions of the Outcomes Study Short Form-36 (SF-36) Health Survey Questionnaire to determine to HRQoL of the students. The VAS using a 10-cm line represented the continuum of the female student's opinion of the degree of pain. One extremity of the line represented ‘unbearable pain’, and the other extremity represented ‘no pain at all’. The participants were asked to rate the degree of pain by making a mark on the line. The scores received from the scale were classified into mild dysmenorrhea if it was between 1–3 points, moderate between 4–7 points, and severe between 8–10 points (16). The MSS system was defined as mild, moderate, and severe based on pain and limited activities, as shown in Table I (14). If an adolescent had pain in the abdominal, groin, and lumbar region on the day before the menstrual period and/or the first day of menstrual period, it was considered to be dysmenorrhea (13).

**Table I.** Verbal multidimensional scoring system for assessment of dysmenorrhea severity.

Severity grading	Working ability	Systemic symptoms	Analgesics
Grade 0: Menstruation is not painful and daily activity is unaffected.	Unaffected	None	None required
Mild (Grade 1): Menstruation is painful but seldom inhibits normal activity; analgesics are seldom required; mild pain.	Rarely affected	None	Rarely required
Moderate (Grade 2): Daily activity is affected; analgesics required and give sufficient relief so that absence from school is unusual; moderate pain.	Moderately affected	Few	Required
Severe (Grade 3): Activity clearly inhibited; poor effect of analgesics; vegetative symptoms (headache, fatigue, vomiting, and diarrhea); severe pain.	Clearly inhibited	Apparent	Poor effect

If an adolescent experienced menstrual bleeding in equal intervals between 21 and 35 days, it was evaluated as regular menstruation (normal); if the menstruation interval was less than 21 days, it was considered to be short; if the menstruation interval was more than 35 days, it was considered to be long. Menstruation of less than 2 days was accepted as short, between 2 and 6 days as normal, and more than 6 days as long.

The SF-36 scale is the most widely used generic instrument for rating HRQoL. The validity and reliability of this instrument has been established for measuring HRQoL in large populations of both healthy and diseased individuals. The original questionnaire was developed by Ware and Sherbourne (17), and reliability and validity studies for the Turkish version of SF-36 were performed by Kocyigit et al. (18). It is a self-evaluation instrument consisting of 36 items which provide assessment in eight domains: physical functioning, social functioning, role limitations due to emotional problems (role–emotional), role limitations due to physical problems (role–physical), bodily pain, vitality, mental health, and general health perception. In our study, we used the Turkish version of SF-36, which showed good reliability and validity in the Turkish validation study (18). The subjects gave appropriate answers for the questions in the SF-36 scale for their depression status during the last 4 weeks. Scores changed from 0 to 100 for each domain separately. The high scores obtained from the scale shows that the HRQoL increases in a positive way.

Following the completion of the questionnaires and inventory, the participants' body mass indexes (BMIs) were calculated by measuring their heights and weights. Each student's body weight was measured with domestic scales and height with a meter rule. Those who had a BMI of 18.0–24.9 kg/m^2^ were classified as normal weight, adolescents with BMI values that corresponded to a BMI of 25.0–29.9 kg/m^2^ were classified as overweight (preobese), adolescents with BMI values that corresponded to an adult BMI of ≥30.0 kg/m^2^ were classified as obese, and adolescents with BMI values that corresponded to <18.0 kg/m^2^ as underweight (19).

In addition, those smoking at least one cigarette a day were evaluated as smokers, those consuming at least 30 g ethyl alcohol in a week as alcohol consumers, those consuming at least 4 glasses of tea in a day (75 cc × 4) as those consuming tea, those consuming at least 3 cups of coffee in a day (150 cc × 3) as those consuming coffee, those consuming at least a glass of cola in a day (200 cc × 1) as those consuming cola, and those eating at least 2 bars of chocolate in a day (150 cc × 3) as those consuming chocolate.

The presence of dysmenorrhea in an adolescent's mother or sister was accepted as a positive family history of dysmenorrhea. Those unemployed, housewife, and retired were considered to be not working. The student allowances were grouped as ‘enough’, ‘medium’, or ‘insufficient’ in the students' own statements. The statistical package for social sciences (SPSS) version 15.0 (Chicago, IL, USA) was used to enter and analyze the data on a personal computer. The statistical analysis was carried out using chi-square, Student's *t* test, backward stepwise logistic regression analysis, and one-way ANOVA test. A value of *P* < 0.05 was considered statistically significant.

### Ethical approval

Permission for the study was obtained by making a petition prior to collecting data. This was achieved by contacting and receiving approval from the Director of the Institution of Eskisehir Osmangazi University. Participants completed an informed consent form in which they were assured of the confidentiality of their responses following which they provided informed verbal consent that participation was voluntary and anonymous. It was also stated that the participant' responses were unidentifiable. All students gave their informed consent prior to their inclusion in the study.

## Results

The average age of the participants was 20.8 ± 1.8 years (range 17–30 years). More than 45.4% of the students (*n* = 283) were in the age group of 20 and below. A total of 8.5% (*n* = 54) of the students were deprived of social health insurance, and 5.1% (*n* = 32) of students reported that their family income status was poor. The proportion of those whose family was of a nucleus type was 88.8% (*n* = 553). The students' prevalence of dysmenorrhea was found to be 72.7% (*n* = 453). In this study, there was no statistically significant difference between those with dysmenorrhea and those without (*P* > 0.05). More detailed socio-demographic characteristics of those with and without dysmenorrhea are shown in Table II.

**Table II.** Some socio-demographic characteristics of students by status of dysmenorrhea.

	Dysmenorrhea			
Socio-demographics	Yes (%)^a^	No (%)^a^	Total (%)^b^	Statistical analysis, chi-square; *P*
Age group (year)				
17–19	14 (74.0)	40 (26.0)	154 (24.7)	0.320; 0.956
20	93 (72.1)	36 (27.9)	129 (20.7)	
21	97 (73.5)	35 (26.5)	132 (21.2)	
22–30	149 (71.6)	59 (28.4)	208 (33.4)	
Social insurance				
Yes	412 (72.3)	158 (27.7)	570 (91.5)	0.400; 0.527
No	41 (77.4)	12 (22.6)	53 (8.5)	
Family income status				
Good	129 (70.1)	55 (29.9)	184 (29.5)	1.857; 0.395
Middle	298 (73.2)	109 (26.8)	407 (65.3)	
Bad	26 (81.3)	6 (18.8)	32 (5.1)	
Family type				
Nuclear	399 (72.2)	154 (27.8)	553 (88.8)	0.549; 0.459
Large	54 (77.1)	16 (22.9)	70 (11.2)	
Total	453 (72.7)	170 (27.3)	623 (100.0)	

^a^Percent for the row. ^b^Percent for the column.

In the study group, the prevalence of smoking cigarettes was 10.3% (*n* = 64), with a 4.8% (*n* = 30) proportion of students consuming alcohol. The number of students who drank four or more cups of tea in a day was 399 (64.0%). Nearly one-third of the students (30.3%, *n* = 189) reported that they consumed at least three cups of coffee in a day; 401 students (64.4%) reported consuming at least one glass of cola a day, and 361 students (57.9%) reported consuming at least two bars of chocolate a day. The mean BMI of the students was 21.05 ± 2.6 kg/m^2^ (min 14.84, max 37.46), and the prevalence of overweight/obesity was 6.4% (*n* = 40). Altogether 54 students (8.7%) reported having a chronic disease necessitating continuous medicine use. There was statistically no difference between habits and medical characteristics of students by status of dysmenorrhea, except for coffee consumption (*P* < 0.001). More detailed habits and medical characteristics of students by status of dysmenorrhea are given in Table III.

**Table III.** Some habits and medical characteristics of students with/without dysmenorrhea.

	Dysmenorrhea			
Some habits	Yes (%)	No (%)	Total (%)	Statistical analysis, chi-square; *P*
Smoking				
Yes	48 (75.0)	16 (25.0)	64 (10.3)	0.082; 0.775
No	405 (72.5)	154 (27.5)	559 (89.7)	
Alcohol consumption				
Yes	23 (76.7)	7 (23.3)	30 (4.8)	0.083; 0.773
No	430 (72.5)	163 (27.5)	593 (95.2)	
Tea consumption				
Yes	291 (72.9)	108 (27.1)	399 (64.0)	0.027; 0.870
No	162 (72.3)	62 (27.7)	224 (36.0)	
Coffee consumption				
Yes	156 (82.5)	33 (17.5)	189 (30.3)	13.205; 0.000
No	297 (68.4)	137 (31.6)	434 (69.7)	
Coke consumption				
Yes	292 (72.8)	109 (27.2)	401 (64.4)	0.006; 0.937
No	161 (72.5)	61 (27.5)	222 (35.6)	
Chocolate consumption				
Yes	273 (75.6)	88 (24.4)	361 (57.9)	3.665; 0.056
No	180 (68.7)	82 (31.3)	262 (42.1)	
Overweight/obese				
Yes	24 (60.0)	16 (40.0)	40 (6.4)	2.831; 0.092
No	429 (73.6)	154 (26.4)	583 (93.6)	
Any chronic disease necessitating the use of any medicine				
Yes	44 (81.5)	10 (18.5)	54 (8.7)	1.833; 0.176
No	409 (71.9)	160 (28.1)	569 (91.3)	
Total	453 (72.7)	170 (27.3)	623 (100.0)	

The students' average menarche age was 13.38 ± 1.20, ranging from 10 to 18. Most students' menarche age was between 13 and 14 (63.2%). About 80% reported experiencing regular menstruation (79.8%).

The average menstrual cycle duration of the students in the study group was 28.73 ± 7.25 days (min 10, max 90), and many students' menstrual cycle duration was between 21 and 34 days (87.5%). Their average menstrual bleeding duration was 5.73 ± 1.34 days, ranging between 3 and 10. Most students' menstrual bleeding duration was less than 7 days (68.7%). Only 8.3% students reported using medicine regulating menstruation. About 50% of students (47.4%) reported having a family history of dysmenorrhea. No difference between menstrual characteristics and status of dysmenorrhea was revealed except for menstrual bleeding duration and family history. The distribution of students with and without dysmenorrhea according to a range of more detailed menstrual characteristics is given in Table IV.

**Table IV.** Some menstrual characteristics of students with/without dysmenorrhea.

	Dysmenorrhea			
Some characteristics	Yes, *n* (%)	No, *n* (%)	Total, *n* (%)	Statistical analysis, chi-square; *P*
Age at menarche (year)				
≤12	106 (79.1)	28 (20.9)	134 (21.5)	3.899; 0.142
13–14	282 (71.6)	112 (28.4)	394 (63.2)	
≥15	65 (68.4)	30 (31.6)	95 (15.3)	
Menstrual regularity				
Regular	359 (72.2)	138 (27.8)	497 (79.8)	0.284; 0.594
Irregular	94 (74.6)	32 (25.4)	126 (20.2)	
Menstrual cycle duration (days)				
≤20	17 (63.0)	10 (37.0)	27 (4.3)	2.135; 0.344
21–34	396 (72.7)	149 (27.3)	545 (87.5)	
≥35	40 (78.4)	11 (21.6)	51 (8.2)	
Menstrual bleeding duration (days)				
≤6	298 (69.6)	130 (30.4)	428 (68.7)	6.565; 0.010
≥7	155 (79.5)	40 (20.5)	195 (31.3)	
Use of medicine regulating menstruation				
Yes	37 (71.2)	15 (28.8)	52 (8.3)	0.010; 0.920
No	416 (72.9)	155 (27.1)	571 (91.7)	
Family history				
Yes	249 (84.4)	46 (15.6)	295 (47.4)	38.619; 0.000
No	204 (62.2)	124 (37.8)	328 (52.6)	
Total	453 (72.7)	170 (27.3)	623 (100.0)	

According to the bivariate analysis results, significant differences were revealed between the existence of dysmenorrhea and the students' consumption of coffee, having a 7-day or more menstrual bleeding duration, and positive family history of dysmenorrhea. Backward stepwise logistic regression analysis performed with the above variables, which showed significantly important findings, is given in Table V. According to this analysis, coffee consumption (OR 2.084), ≥7 days menstrual bleeding duration (OR 1.590), and family history (OR 3.043) were significantly important risk factors for dysmenorrhea.

**Table V.** Significant independent variables for dysmenorrhea according to logistic regression analysis.

Variables	β	SE	*P*	OR	95% CI
Constant	0.202	0.137	0.411		
Coffee consumption (reference: no)					
Yes	0.734	0.224	0.001	2.084	1.342–3.235
Menstrual bleeding duration (days) (reference: ≤6)					
≥7	0.464	0.214	0.031	1.590	1.044–2.421
Family history (reference: No)					
Yes	1.113	0.200	0.000	3.043	2.058–4.500

Hosmer and Lemeshow test: chi-square = 3.616; *P* = 0.606.

There was no difference between the average scores that students with and without dysmenorrhea received from HRQoL scale, with the exception of the domains of physical functioning (*P* < 0.05), role–physical (*P* < 0.05), bodily pain (*P* < 0.001), general health perception (*P* < 0.05), and vitality (*P* < 0.05). In those with dysmenorrhea the scores received from all the domains of HRQoL were lower. More detailed data received from SF-36 domains by status of dysmenorrhea are given in Table VI.

**Table VI.** Average scores students received from SF-36 domains by status of dysmenorrhea.

	SF-36 score		
	Dysmenorrhea		Statistical analysis
Domains	Yes (*n* = 453) (mean ± SD)	No (*n* = 170) (mean ± SD)	*t* test; *P-*value
Physical functioning	81.53 ± 17.85	84.87 ± 16.74	2.115; 0.035
Role–physical	77.37 ± 33.86	84.71 ± 29.57	2.490; 0.013
Bodily pain	60.01 ± 18.73	69.41 ± 20.34	5.450; 0.000
General health perception	56.02 ± 17.78	60.34 ± 17.91	2.696; 0.007
Vitality	52.16 ± 16.13	55.77 ± 16.28	2.476; 0.014
Social functioning	69.29 ± 20.62	72.28 ± 20.87	1.607; 0.108
Role–emotional	53.57 ± 42.65	59.80 ± 42.74	1.624; 0.105
Mental health	57.36 ± 16.62	59.03 ± 17.04	1.110; 0.267

The average scores that students received from the SF-36 scale by severity of dysmenorrhea are given in Table VII. The distribution of the severity of dysmenorrhea cases was as follows: mild 33.8% (*n* = 153), moderate 42.4% (*n* = 192), and severe 23.8% (*n* = 108).

**Table VII.** Connection between the severity of dysmenorrhea and mean scores of SF-36 domains.

	Severity of dysmenorrhea			
SF-36 Domains	Mild (*n* = 153) (mean ± SD)	Moderate (*n* = 192) (mean ± SD)	Severe (*n* = 108) (mean ± SD)	Statistical analysis F test; *P*-value
Physical functioning	84.10 ± 16.68	80.93 ± 17.17	78.96 ± 20.18	2.830; 0.060
Role–physical	86.11 ± 26.73	74.22 ± 34.88	70.60 ± 38.49	8.353; 0.000
Bodily pain	65.05 ± 17.46	60.13 ± 17.61	52.66 ± 20.11	14.695; 0.000
General health perception	59.03 ± 16.94	55.64 ± 17.90	52.44 ± 18.15	4.499; 0.012
Vitality	56.11 ± 14.91	50.42 ± 16.42	49.68 ± 16.35	7.185; 0.001
Social functioning	72.79 ± 20.13	66.60 ± 21.45	69.10 ± 19.22	3.894; 0.021
Role–emotional	58.39 ± 42.80	48.79 ± 43.40	55.25 ± 40.55	2.281; 0.103
Mental health	60.59 ± 16.48	55.87 ± 16.99	55.43 ± 15.62	4.450; 0.012

## Discussion

The present study found a high prevalence of dysmenorrhea (72.7%) reported among female students; this figure is consistent with previous studies reporting rates between 28% and 89.5% (4–7). Similarly, previous studies conducted in Turkey indicated that the prevalence of dysmenorrhea among the same age group of women ranged between 23.4% and 89.5% (20–22). A reason for the variation in these estimates may be the use of selected groups of women, and the absence of a universally accepted method of defining dysmenorrhea, which was probably as greatly responsible for the disparity as the methods of collecting data, the study definitions of dysmenorrhea and pain, and the study populations themselves (23).

In this survey, approximately two-thirds of the women (66.2%) described their dysmenorrhea as severe and moderate, in line with the study by Burnett et al. (4). This indicates that dysmenorrhea is still an important public health problem and that these female students in rural areas experience severe or moderate dysmenorrhea, which may have a negative effect on HRQoL, social environment, work, and psychological status.

Many studies determined that the prevalence of dysmenorrhea showed a decrease with increasing age, indicating that primary dysmenorrhea peaks in late adolescence and the early 20s and the incidence falls with increasing age (4,13,24). However, this study did not find any connection between age groups and the prevalence of dysmenorrhea (*P* > 0.05). This is probably because the students in the study group may not be in a higher range of years.

Epidemiological studies (25) have shown a link between dysmenorrhea and several environmental risk factors, including current cigarette smoking. However, this study did not find any relationship between cigarette use and dysmenorrhea (*P* > 0.05). One explanation for this could be that in this study we did not ask the dose of cigarettes used and the period of smoking, in line with another study from Turkey (24).

By both univariate and multivariate analyses, coffee consumption was an important risk factor for dysmenorrhea, in line with some researchers' studies (26), and similarly menstrual bleeding duration of 7 days and over was an important risk factor for dysmenorrhea. This finding is compatible with the result showing that the risk of dysmenorrhea is higher in women with long menstrual flows (27).

According to the bivariate and multivariate analysis, those with family history of dysmenorrhea had a significantly higher prevalence of dysmenorrhea, a finding which is consistent with some studies (7). This result indicates that a family history of dysmenorrhea seems to be an important characteristic for women with dysmenorrhea. As an explanation for this, some researchers have reported that daughters of mothers who have menstrual complaints also experienced menstrual discomfort, and that the reason for this could be related with behavior that is learned from the mother (7,10). The fact that family history was shown to be a risk factor for dysmenorrhea may be related to the risk for related conditions such as endometriosis, which has already been shown to have a familial pattern (13).

In this study, the scores received from many of the SF-36 domains (physical functioning, role–physical, bodily pain, general health perception, and vitality) were significantly lower in students with dysmenorrhea. An explanation for why only some but not all of the parameters are affected could be that since dysmenorrhea is an important illness that primarily affects physical health, domains of the SF-36 related to physical health could only be affected. Social and psychological parameters such as social functioning, role–emotional, and mental health could not be affected due to this fact.

In addition, with the increasing severity of dysmenorrhea, the average scores received from all the domains of SF-36 showed decrease, consistent with the study by Barnard et al. (28), indicating that women with dysmenorrhea and the other menstrual symptoms had lower HRQoL values.

The prevalence of dysmenorrhea among female students was relatively high throughout our study, reaching almost three-quarters (72.7%), and HRQoL showed a decrease in the presence of dysmenorrhea and with the increase in the severity of dysmenorrhea.

We are well aware of the limitations of the present study. Firstly, it was performed in a single district, and in a single university, therefore the sample may not be representative of all Turkish female university students. In other words, its comparability with community-based studies is weak, because the mean age of the female students in the study group was rather low compared to that of the general population. In addition, when taking into consideration that dysmenorrhea decreases with increasing age, its being done with a determined age group hinders its applicability to all women. Secondly, a further limitation is that this study was a cross-sectional study, thus precluding inferences of causality among variables. The last limitation is that the nature of self-reporting may have resulted in under-reporting of the conditions.
